# Transcriptional Profiling of Human Brain Endothelial Cells Reveals Key Properties Crucial for Predictive *In Vitro* Blood-Brain Barrier Models

**DOI:** 10.1371/journal.pone.0038149

**Published:** 2012-05-31

**Authors:** Eduard Urich, Stanley E. Lazic, Juliette Molnos, Isabelle Wells, Per-Ola Freskgård

**Affiliations:** 1 CNS Research, F. Hoffmann-La Roche Ltd, Basel, Switzerland; 2 Bioinformatics and Exploratory Data Analysis, F. Hoffmann-La Roche Ltd, Basel, Switzerland; 3 Translational Research Science, F. Hoffmann-La Roche Ltd, Basel, Switzerland; Massachusetts General Hospital/Harvard Medical School, United States of America

## Abstract

Brain microvascular endothelial cells (BEC) constitute the blood-brain barrier (BBB) which forms a dynamic interface between the blood and the central nervous system (CNS). This highly specialized interface restricts paracellular diffusion of fluids and solutes including chemicals, toxins and drugs from entering the brain. In this study we compared the transcriptome profiles of the human immortalized brain endothelial cell line hCMEC/D3 and human primary BEC. We identified transcriptional differences in immune response genes which are directly related to the immortalization procedure of the hCMEC/D3 cells. Interestingly, astrocytic co-culturing reduced cell adhesion and migration molecules in both BECs, which possibly could be related to regulation of immune surveillance of the CNS controlled by astrocytic cells within the neurovascular unit. By matching the transcriptome data from these two cell lines with published transcriptional data from freshly isolated mouse BECs, we discovered striking differences that could explain some of the limitations of using cultured BECs to study BBB properties. Key protein classes such as tight junction proteins, transporters and cell surface receptors show differing expression profiles. For example, the claudin-5, occludin and JAM2 expression is dramatically reduced in the two human BEC lines, which likely explains their low transcellular electric resistance and paracellular leakiness. In addition, the human BEC lines express low levels of unique brain endothelial transporters such as Glut1 and Pgp. Cell surface receptors such as LRP1, RAGE and the insulin receptor that are involved in receptor-mediated transport are also expressed at very low levels. Taken together, these data illustrate that BECs lose their unique protein expression pattern outside of their native environment and display a more generic endothelial cell phenotype. A collection of key genes that seems to be highly regulated by the local surroundings of BEC within the neurovascular unit are presented and discussed.

## Introduction

The specific microenvironment of the central nervous system (CNS) is vital for proper neuronal function. A key feature which provides and maintains the extracellular medium compatible with normal neuronal activity is the blood brain barrier (BBB). Consequently, the failure of BBB structural integrity and function plays a pivotal role in the pathogenesis of many diseases of the CNS [1], [2]. The anatomical constituents of the BBB are the specialized brain endothelial cells (BECs) that together with pericytes, astrocytes, neurons, and possibly other glial cells, comprise the neurovascular unit (NVU) [3], [4], [5], [6]. Although all these cell types contribute to the functioning of the brain microvasculature, only the BECs are thought to control permeability directly or indirectly via stimuli from the other cells in the NVU [7], [8].

While the BBB performs an important function in keeping out unwanted or harmful molecules from the brain, it poses a challenge for delivering valuable therapeutics such as anticancer, antibiotic, neuroprotective or antipsychotic drugs into the brain. Consequently, finding beneficial molecules that also cross the BBB is an increasing problem within the pharmaceutical industry, especially if these molecules are large biotherapeutics such as proteins and antibodies. There is a growing need for reliable bench models that predict important *in vivo* properties. These models would facilitate our understanding of key biological functions of the BBB and allow study of specific transport systems potentially suitable for delivering drugs to the brain. Pharmaceutical research in particular is dependent on well characterized and easy to handle *in vitro* models. Therefore, BEC culture models have been developed for the study of the BBB with an attempt to mimic important *in vivo* properties. Over the last few years, the isolation and culture of BEC have advanced significantly, resulting in a variety of BBB models.

Endothelial cells (ECs) from various brain regions and species have been used. The cells have been isolated by different techniques and variation in subsequent culturing procedure of the BECs has also been evaluated [9], [10]. One limitation of cell cultures is their potential dedifferentiation behavior where the cells lose properties they originally inherently possessed due to the lack of the natural *in vivo* environment [11], [12], such as gene expression patterns and certain functionalities. Therefore, attempts have also been made to impose BBB properties on BECs by co-culturing them with astrocytes or pericytes [13] or by using sheer stress [14]. This has in many cases improved some of the properties of the BECs, such as increased restriction in paracellular leakiness and elevated transendothelial electric resistance (TEER). Unfortunately only a limited number of proteins and a few properties have been studied in cultured BECs, meaning that our basic understanding of what regulates BEC properties is very restricted. Furthermore our knowledge about the global expression pattern in cultured BECs is still very incomplete.

The aim of this study was to increase our understanding of gene expression patterns which are required for a well-functioning *in vitro* BBB model. This was performed by quantifying the global gene expression profile in the hCMEC/D3 cell line and in primary human BEC (hpBEC). The hCMEC/D3 has been used and characterized extensively to study BBB properties *in vitro*, so this cell line functioned as a good reference for a cellular BEC model [15]. The hCMEC/D3 cell line was generated by immortalization of primary human brain capillary endothelial cells via a lentiviral vector system. The hpBEC was included as a model system for primary brain endothelial cells that could potentially demonstrate important differences compared to a stable cell line. The hCMEC/D3 cell line has been shown to express typical endothelial cell markers, such as CD31, VE-cadherin and von Willebrand factor, to show a stable karyotype, to preserve contact inhibition for monolayers in culture and to form capillary tubes in matrix [15]. However, the cells show deficiency in typical and important brain endothelial properties such as low TEER value and relative high permeability towards small tracer molecules indicating paracellular leakiness and suboptimal formation of tight junctions (TJs). In our hands, similarly poor functional properties were observed for the hpBEC regardless of their primary source also indicating issues with appropriate TJ formation.

In order to understand these suboptimal functional properties a global transciptome analysis was performed on these two BEC lines. The transcription profile was compared with a recently published analysis of freshly isolated mouse brain BECs [16]. A recent cross-species analysis showed that gene expression is significantly preserved between the two species [17], supporting the comparisons of human and mouse transcriptome data. The comparison clearly showed that critical genes reported to be responsible for structural and functional properties of the TJ are expressed at very low levels in both cell lines. Two key genes specifically found in BECs, claudin-5 and occludin, are expressed at very low levels, not only in comparison to the mouse data but also in comparison to other TJ genes. We also found major differences in the SLC and ABC transporter families. In particular, family members that are known to be characteristic of BECs, such as Glut1, Pgp, MRP4 and BCRP, are expressed at very low levels based on a similar comparison. In addition, the analysis also indicates major differences in the expression pattern of a collection of important cell surface receptors, which also have direct implications for the study of transport mechanisms. Genes altered due to the immortalization procedure are also identified and are linked to the immune and interferon pathways. This analysis clarifies many of the atypical BBB properties of the BEC lines and can be explained by this gene expression analysis which has generated essential information to further improve in vitro BBB models. One interesting observation is the specific down-regulation of adhesion molecules on the BECs in the presence of astrocytes. This might be linked to a novel function of astrocytes in regulating cell adhesion and, indirectly, immune surveillance of the CNS.

Taken together, our data strongly indicate that brain ECs lose their unique protein expression pattern outside their native *in vivo* environment resulting in a more generic EC phenotype. Our findings indicate that specific transcription of genes in brain ECs are at least in part dictated by other cells within the NVU.

## Results

### Different endothelial marker expression and growth behavior of the hCMEC/D3 and hpBEC cells

**Figure 1:**
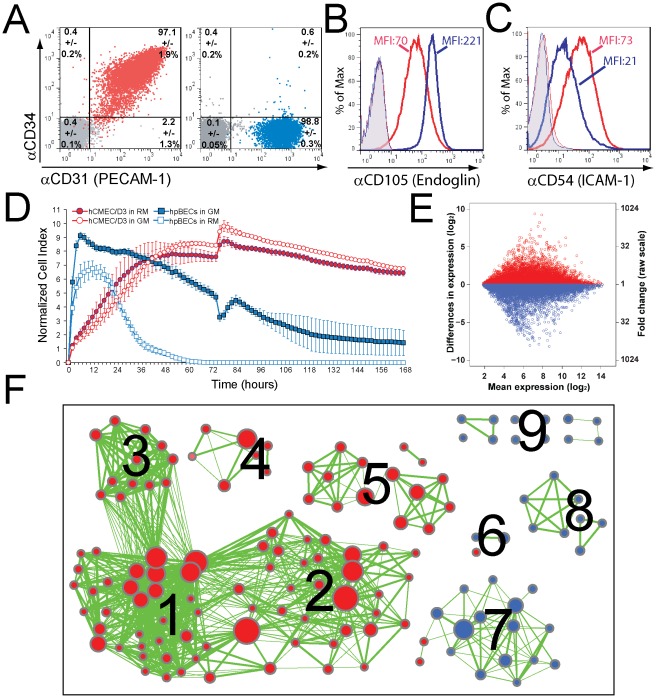
Immortalization influences BEC phenotype, growth behavior and expression of cell division related genes. *(* ***A–C*** *)* Flow cytometry analysis of confluent hCMEC/D3 (red population, red histograms) and hpBECs (blue population, blue histograms) seeded on collagen I coated inserts. Staining was done with indicated antibodies or relevant isotype controls (grey population, tinted histograms). The average population sizes and standard deviations of three similar experiments are shown in **A**, whereas one representative experiment of three is shown in ***B & C*** (MFI = mean fluorescence intensity). *(* ***D*** *)* Real time monitoring of adherent BECs cultured in Resting (RM) or Growth Medium (GM) by the xCeLLIgence System. The curves show the time-, attachment- and density-dependent cell growth and viability of the individual BEC lines respectively culturing conditions. *(* ***E*** *)* Comparison of gene expression between hCMEC/D3 cells and hpBECs. The data is represented as a dot plot on a log_2_ scale, where each point represents a probe set on the gene chip. Red and blue dots indicate probe sets, which have higher expression in hCMEC/D3 (red) or higher expression in hpBECs (blue). The mean expression values are averaged expression values for both cell lines. *(* ***F*** *)* The enrichment map displays the differently expressed gene sets between the two BEC lines. Red node color represents higher expression in hCMEC/D3 cells, whereas blue represents higher expression in hpBECs. Node size is proportional to the number of genes in the gene set and edge thickness represents the degree of overlap between two gene sets. Labels for the clusters of functionally related gene sets were manually assigned: 1) Mitosis, 2) DNA Repair, 3) Anaphase Promoting Complex, 4) Immune and Virus Response, 5) RNA Processing, 6) Cell-Cell Adhesion, 7) Differentiation/Maturation/Development, 8) Metabolic Processes, 9) Miscellaneous.

Flow cytometry analysis (FACS) for CD31 (**Figure 1a**), CD34 (**Figure 1a**), CD105 (**Figure 1b**) and CD54 (**Figure 1c**) of hpBECs and hCMEC/D3 cells confirmed their endothelial identity [15], [18] and purity, but also revealed some differences between these BEC lines. The surface expression of CD31 on both cell lines is similar, whereas the hpBECs are negative for CD34 (**Figure 1a**). In comparison to the hCMEC/D3, the hpBECs express more CD105 (**Figure 1b**) but lower levels of CD54 (**Figure 1c**). This is in agreement with the transcriptional data where the hCMEC/D3 cells had log_2_ values of 11.78 for CD31, 9.21 for CD34, 9.97 for CD105 and 8.13 for CD54. The hpBECs cells on the other hand had log_2_ values of 10.11 for CD31, 6.42 for CD34, 10.83 for CD105 and 8.87 for CD54 (**Table S1**). In addition, phosphorylation of the endothelial cell-specific receptor tyrosine kinase TIE2 [16], [19] following stimulation with 1 mM pervanadate was also confirmed positive on both cells (97.8% of the hpBECs & 98.4% of the hCMEC/D3) (**data not shown**).

The dynamic monitoring of the hCMEC/D3 growth and viability, using the xCelligence RT-CA system [20], [21], revealed some large differences compared to the hpBECs (**Figure 1d**). The interaction of adherent mammalian cells with the microelectrodes of the xCelligence RT-CA system leads to an impedance change that is proportional to the cell number and morphology as well as the quality of cell attachment [20], [21]. Our data show that the hpBECs are unable to form a stable confluent monolayer over an extended period and show sensitivity to media components of the Growth Medium (EGM-2 supplemented EBM-2 Medium, see Material and Methods) (**Figure 1d**). After a log growth phase, the hCMEC/D3 cells reach a plateau that persists for several days without major fluctuations. The use of Resting Medium lacking the endothelial growth factors gave a slightly higher cell index (CI) for the hCMEC/D3 during their plateau phase which was not advantageous for the hpBECs (**Figure 1d**). The morphological and proliferative characteristics are confirmed by phase contrast microscopy (**Figure S1**). In addition to the difference in cell size, we observed that based on cell number calculations the hCMEC/D3 are 5-fold significantly tighter packed then the hpBECs 3 days post seeding(p<0.001) . Freshly plated hCMEC/D3 typically grew to confluence within 2–3 days. Light microscopy of the monolayer at 3 days post seeding revealed a characteristic elongated spindle shaped morphology of the cells (**Figure S1**) typical for primary or low passage microvascular endothelial cultures. However, we observed that culturing the hCMEC/D3 cells for more than 7 days after confluence (or 10 days after cell seeding) resulted in cell overgrowth on the transwell filters (**data not shown**).

### Gene expression differences between immortalized and primary BECs

We assessed the overall gene expression profile for the immortalized hCMEC/D3 and primary hpBEC cell lines using published standard growing conditions (**see Material & Methods**). A total of 21460 probe sets out of 38172 high quality probe sets (56%) surveyed by microarray met the criteria to be considered expressed (i.e. hybridization signal above cutoff ≥5 for at least 2 of 16 samples (**Table S1**). The coefficient determination (R^2^) for mRNA levels between the cell lines was 0.912 (p<1.68 ⋅ 10^−6^), meaning that 91% of the variance in gene expression of one cell line is accounted for by levels of gene expression in the other cell line. Pairwise comparison between the immortalized hCMEC/D3 versus primary hpBECs revealed a large proportion of probe sets that were significantly differently expressed (≥10-fold = 2% of all high quality probe sets). This limit (≥10-fold) led to the identification of 148 and 272 genes as higher or lower expressed, respectively, in the hCMEC/D3 cells (**Table S1**). This is graphically represented by a dot plot where each dot represents a probe set on the Affymetrix GeneChip and its position relative to the log_2_ (**Figure 1e**). The axes represent expression levels in the different cell types. Gene set enrichment analysis was performed using gene ontology (GO) biological process gene sets. Only gene sets passing conservative significance thresholds (p≤0.05) are displayed in the enrichment map (**Figure 1f**) resulting in 154 GO categories which differed between the cell types (**Table S2**). The enrichment map visualizes functionally coherent gene sets [22], in which gene sets are organized into a similarity network, where nodes represents gene sets and weighted links between the nodes represent the overlap of member genes [23]. Most of the higher-expressed genes in hCMEC/D3 were involved in DNA repair, RNA processing, mitosis and immune/virus response (**Figure 1f** **, Table S1 & S2**). This is not completely unexpected since the hCMEC/D3 cells have been immortalized by co-expression of human telomerase reverse transcriptase (hTERT) and the Simian virus (SV40) large T antigen (TAg) [15]. SV40 TAg is a powerful viral oncoprotein capable of transforming a variety of cell types, leading to expanded proliferation and survival potentials [24], [25], [26]. The majority of the higher-expressed genes in the hpBECs can be related to catabolic/metabolic processes, vesicle transport, endothelial cell migration and differentiation/maturation (**Figure 1f** **, Table S1 & S2**).

### Interferon related genes up regulated in hCMEC/D3 cells

**Figure 2:**
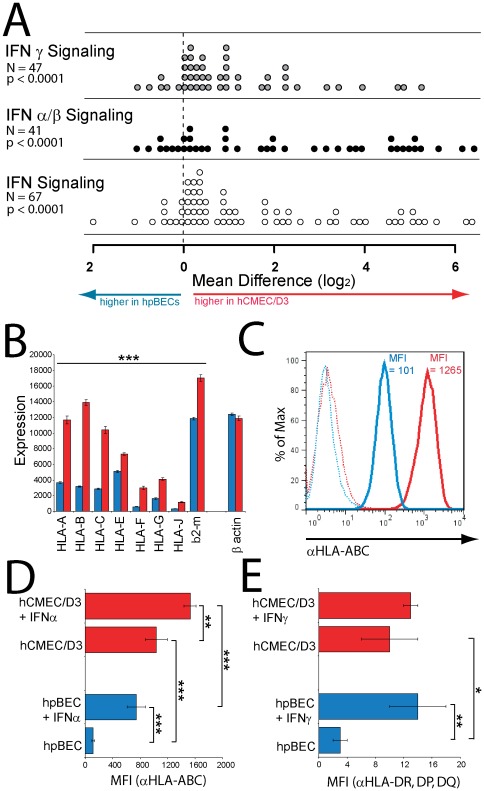
Induction of INF-stimulated genes in immortalized BECs. *(* ***A*** *)* Expression levels of total IFN, IFNα/β and IFNγ signaling pathway genes were determined by microarray analysis for the two BEC lines. Each circle represents one gene of the indicated reactome pathway and the difference in expression between hCMEC/D3 and hpBEC cells. To compare IFN signaling reactomes between BEC lines, the values of the four replicates were averaged, and the probe-set with the highest value was used to represent each gene. The distributions of the genes within each gene set were compared with a one-sample t-test, to test whether the mean of the distributions were different from zero. *(* ***B*** *)* HLA class I, b2-microtublin and β actin gene expression in resting hCMEC/D3 (red bars) and hpBECs (blue bars) determined by microarray analysis. All displayed HLA class I and b2-microtublin genes were significantly (p<0.001) higher expressed in the hCMEC/D3 cells. *(* ***C–E*** *)* Flow cytometry analysis of surface expression of *(* ***C & D*** *)* HLA class I and *(* ***E*** *)* II molecules on hCMEC/D3 cells (red histograms, red bars) and hpBECs (blue histograms, blue bars). *(* ***C*** *)* The HLA class I expression levels and the mean fluorescence intensities (MFI) on both resting BEC lines, in one of four similar experiments, is presented. *(* ***D*** *)* The effect of IFNα upon HLA class I surface expression and *(* ***E*** *)* the effect of IFNγ upon HLA class II surface expression on the two BEC lines are displayed as average MFIs of three similar experiments. *p<0.05, **p<0.01 and ***p<0.001 (Student's *t*-test).

By comparing the most differently expressed genes in the hCMEC/D3 with those in the hpBECs we found that many of them are linked to viral infection and can be assigned to the interferon (IFN) signaling pathway, as defined by the Reactome database [27] (**Table S1**). Among those 132 genes which are 10-fold or more expressed in the hCMEC/D3 cells compared to the hpBECs, 13% belong to the IFN signaling pathway. By comparing the expression levels of the 64 INF signaling genes present on the arrays between the cell types, we can see a significant increase of expression (p<0.0001) in the hCMEC/D3 (**Figure 2a** **& Table S3**). A comparable picture was seen for INFα/β and INFγ pathways (**Figure 2a**). While the transcription of the IFN-stimulated genes (ISGs) is enhanced in the hCMEC/D3 cells, the transcription of the IFNs, IFN-receptors and IFN associated signaling molecules are comparably high in both cell types (**Table S3**). Exposure of human cells to viral proteins induces the production IFNs. Therefore the IFN system is the first line of viral defense and a powerful antitumor response in vertebrates [28], [29]. Recent evidence suggests that many of the ISGs are indeed induced by SV40 TAg [30] and affect many aspects of cellular physiology [31]. INFα/β exert their antiviral and antitumor effects through mechanisms that include the induction of MHC class I molecules (MHC I) expression on the cell surface of infected or oncogenic transformed cells [32], [33], [34], [35], [36]. IFNγ, on the other hand, increases both MHC I expression and leads to a *de novo* expression of MHC class II (MHC II) molecules [37]. In fact we observed a significantly enhanced expression of all MHC I (**Figure 2b** **& Table S1**) and certain MHC II haplotypes (HLAs) (**Table S1**) in the hCMEC/D3 cells versus the hpBECs on transcriptional level. This observation could be validated at protein level by FACS staining of MHC I HLA-A,B,C (**Figure 2c & 2d**) and MHC II HLA-DR,DP,DQ (**Figure 2e**) between the cell types, in which the hCMEC/D3 cells showed a 10-fold higher basal surface expression of HLA-A,B,C (**Figure 2c & 2d**) and a 4-fold higher basal surface expression of HLA-DR,DP,DQ (**Figure 2e**). The HLA-A,B,C basal surface expression on HUVECs showed similar MFI (MFI = 112±9) to the pBECs. The effects of IFNα and IFNγ stimulation upon BECs surface expression of HLA-A,B,C and HLA-DR,DP,DQ were also examined by FACS quantification (**Figure 2d & 2e**). As can be seen in **Figure 2d** IFNα stimulation caused a 6-fold increase in hpBECs and a doubling of HLA-A,B,C surface expression in hCMEC/D3. Similarly, IFNγ stimulation caused a 5-fold increase in hpBECs and a non-significant doubling of HLA-DR,DP,DQ surface expression in hCMEC/D3 cells (**Figure 2e**). The data suggest that the hCMEC/D3 is already in a stimulated state with high expression of MHC molecules on the cell surface which is likely to be caused by the immortalization procedure using SV40 TAg.

### Astrocytic co-culturing modulates the immune state of the BECs

**Figure 3:**
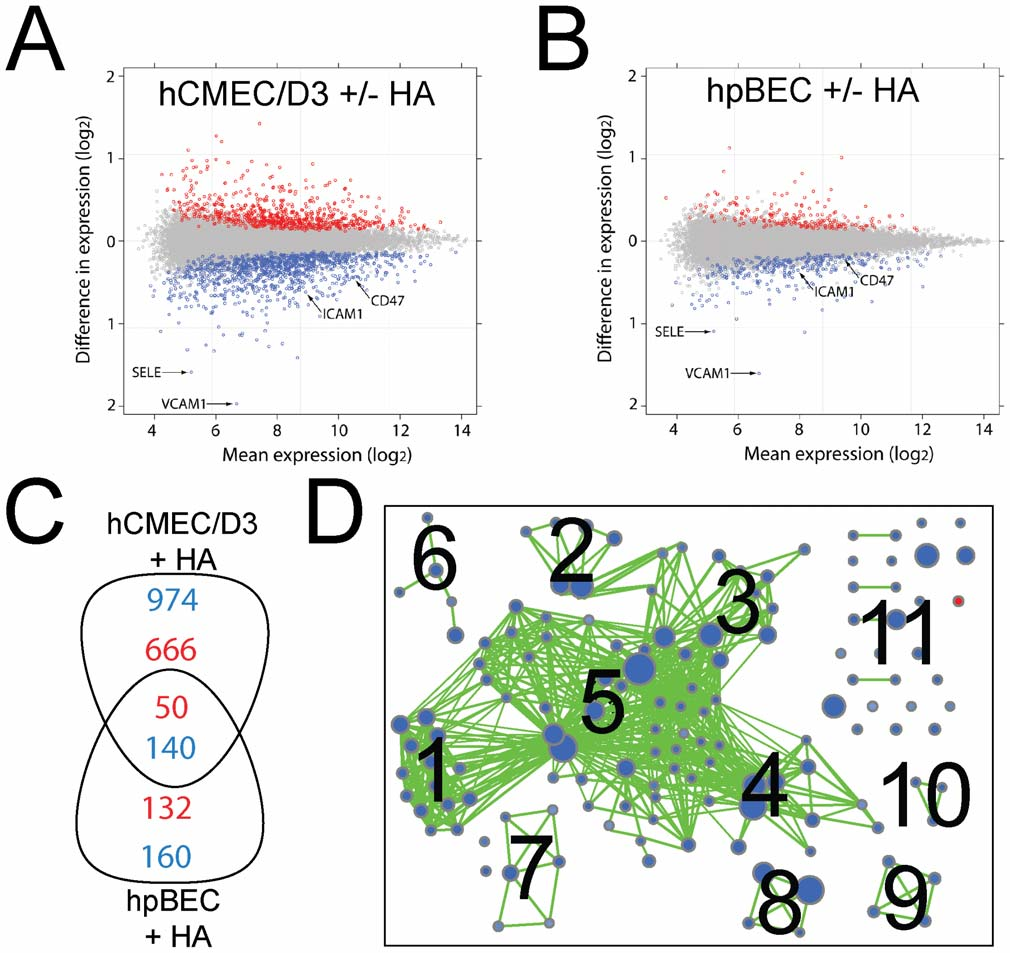
Astrocytic co-culturing reduced the expression of adhesion molecules. The expression of genes in *(* ***A*** *)* hCMEC/D3 and *(* ***B*** *)* hpBECs was compared in presence and absence of human astrocytes (HA). The data is represented as a dot plot on a log_2_ scale, where each point represents a probe set on the gene chip. Red and blue dots indicate probe sets, which are differently expressed (adjusted p≤0.05) between culturing conditions. Blue dots represent lower and red dots higher expressed probes in the co-culturing conditions with HAs versus culturing the BECs alone. The mean expression values are averaged expression values for both cell lines. *(* ***C*** *)* Overlapping genes identified between the two BECs in the co-culturing conditions with HAs. The numbers of up-regulated (red numbers) or down-regulated (blue numbers) genes in the BECs with HA co-culture are displayed. *(* ***D*** *)* The enrichment map displays the differently expressed gene sets for hCMEC/D3 cells between culturing conditions. Blue node color represents lower expression in hCMEC/D3 + HA, whereas red represents higher expression in hCMEC/D3 control. Node size is proportional to the number of genes in the gene set and edge thickness represents the degree of overlap between two gene sets. Labels for the clusters of functionally related gene sets were manually assigned: 1) Regulation of Immune Cell Activation and Proliferation, 2) Regulation of Kinase Cascade, 3) Regulation of Inflammatory and Defense Response, 4) Response to Pathogens, 5) Regulation of the Immune System, 6) Response to Cytokines, 7) Regulation of Cell-Cell Adhesion, 8) Cell Migration, 9) Signal Transduction, 10) Antigen Processing and Presentation, 11) Miscellaneous.

Contact co-culture with astrocytes has been reported to restore some of the dedifferentiated BBB phenotype of isolated BECs [38], [39], [40] by having a particular impact on the expression and maintenance of the tight junction (TJ) proteins [38], [40], [41], [42], [43], [44], [45]. To investigate if secreted astrocytic components could influence the gene expression of the hCMEC/D3 and hpBEC cells, the cells were co-cultured with astrocytes and transcriptome data was collected. The phenotypic status of the astrocytes was verified using FACS analysis (**Figure S2**). Numerous genes were significantly affected, but the change in expression levels was small indicating low responsiveness towards astrocytic factors (**Figure 3a & 3b**). Only 8.5% (1830 genes) in the hCMEC/D3 cells and 2.2% (482 genes) in the hpBECs of all 21460 expressed genes (i.e. mean hybridization signal above cutoff ≥5) were differentially expressed (adjusted p-values<0.05) between the control and the co-culturing conditions (**Figure 3a & 3b**). Thus, the vast majority of gene expression was not altered in an environment containing astrocytes (**Figure 3a & 3b**). Proteins belonging to the TJ family were not affected by the presence of astrocytes (**Table S1 & S4**). Since we chose non-contact co-culture conditions in order to avoid contamination by astrocytic mRNA, this set up might explain the small gene expression changes and indicate the importance of cell-cell interaction through the astrocyte end-feet structures for the TJ protein expression. However, genes that were significantly decreased in both BECs in the presence of astrocytes could be assigned to cell-cell adhesion, cell extravasation, immune response, response to other organisms and cell migration (**Figure 3c** **& Table S4**). In particular, the two genes most reduced by astrocytic co-culturing in both cells were E-selectin and VCAM-1 (**Figure 3a & 3b**). These two cell adhesion molecules are involved in leukocyte recruitment and migration across the BBB [46], [47]. FACS staining for ICAM-1 on hCMEC/D3 cells confirmed the astrocytic effect on ECs adhesion molecule expression (**Figure S3**). As a potent source of immunologically relevant cytokines and chemokines [48], [49] it is conceivable that astrocytes may modulate the expression of adhesion molecules that play a key role in maintaining the immunologically privileged status of the brain. Genes that were specifically affected by astrocytic co-culturing in both BECs are summarized in **Table S5** and gene sets which were affected by astrocytic co-culturing only in one of the two cell lines are displayed in **Table S4**. A gene set enrichment analysis for hCMEC/D3 cells was performed. Only gene sets passing conservative significance thresholds (p≤0.05) are displayed in the enrichment map (**Figure 3d**) resulting in 141 GO categories which differed between controls and HA non-contact co-culturing condition (**Table S6**). Almost all significant gene sets were lower expressed in the HA co-culture condition and can be mostly related to immune system regulation (**Figure 3d** **& Table S6**).

### Expression of proteins important for tight junction formation

**Figure 4:**
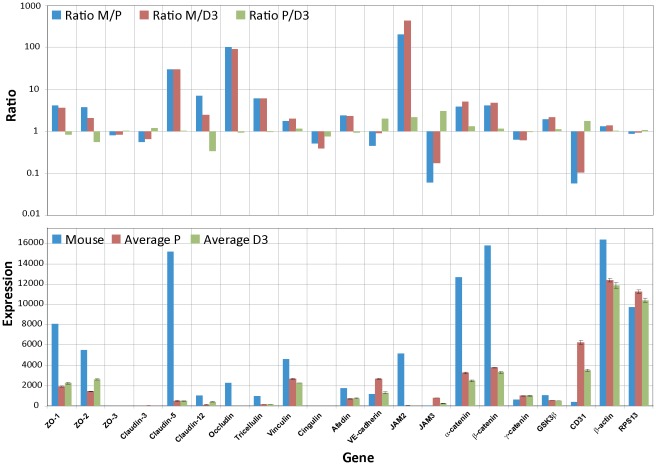
Differences in expression levels of TJ protein between cultured BECs and freshly isolated BECs. The RPL4 normalized mouse expression values (Mouse) are compared to the also RPL4 normalized expression values of hpBECs (Average P) and hCMEC/D3 (Average D3). The lower graph shows the absolute expression levels for each cell type. The upper graph shows the ratio between RPL4 normalized mouse BECs and hpBECs (Ratio M/P); the ratio between RPL4 normalized mouse BECs and hCMEC/D3 (Ratio M/D3) and the ratio between hpBECs and hCMEC/D3 (Ratio P/D3). Three genes that are expressed in much lower levels in both the hpBECs and the hCMEC/D3 cell line are claudin-5, occludin and JAM2. Two genes that are expressed at higher levels in the human cell lines are JAM3 and CD31.

One of the most distinct characteristics of BECs is the presence of highly organized TJs. These TJs are responsible for the selective permeability towards large and small molecules and the high TEER value. Transmembrane proteins of TJ include occludin, claudins and junctional adhesion molecules (JAM). These proteins interact with cytosolic proteins such as occludens proteins (ZO) which are associated with the actin network of the cell cytoskeleton [50]. By specifically examining the expression of these proteins, it could be shown that claudin-5, β-catenin and ZO-1 are present at a certain level in the hCMEC/D3 cells and are expressed predominantly at the junctions between cells [15]. However, a completely different picture emerged when the transcriptional profiles were analyzed and compared to the mouse data obtained from freshly isolated BECs [16]. The data clearly show that three TJ specific genes, claudin-5, occludin and JAM2, are drastically reduced in expression level in both cell lines compared to the mouse cells (**Figure 4**). Claudin-5 has been shown to play an important role in preventing small (<800 D) but not large molecules crossing the BBB [51]. Furthermore, it has been shown that the ratio between claudin-5 and -12 seems to be important for proper TJ formation. Claudin-5 was 751-fold more expressed compared to claudin-12 in rats [52]. A similar ratio was also found using primary mouse BECs, where the mRNA expression levels of claudin-5 was 28-fold higher than claudin-12 [16]. Our analysis of the human primary and the immortalized BECs shows a much smaller difference between claudin-5 and claudin-12. In hCMEC/D3 cells the expression levels for these two claudins were almost equal (**Figure 4**). Other proteins which are also less expressed in both cultured BEC lines compared to the freshly isolated mouse BECs [16] are ZO-1, ZO-2, claudin-12, tricellulin, α- and β-catenin (**Figure 4**). They have also been described to be important for the BBB tightness [53]. Interestingly, claudin-3 and ZO-3 are expressed at low levels in both cultured cell lines as well as in the fresh preparation of mouse BECs. This could point to a less important role for these two proteins in maintaining the BBB integrity. One of the proteins which is for example higher expressed in the cultured cell lines compared to the fresh *ex vivo* mouse BECs is CD31, which is not surprising since CD31 was the selection marker used during the isolation, characterization and immortalization process of the hCMEC/D3 cells [15].

The comparison between the human cell lines and the mouse data was performed by normalizing the expression levels to the described housekeeping gene ribosomal protein L4 (RPL4) which has been shown to be one of the top internal control genes for cross-species comparison [54]. The procedure was confirmed by comparing the normalized expression values for two other known housekeeping genes, actin and the ribosomal protein S13 showing that these genes are expressed at very similar levels in the cultured human BEC lines and the freshly prepared mouse BECs (**Figure 4**). In addition, all proposed housekeeping genes were also analyzed and show only minor variations between species (**Figure S4**) demonstrating that any of the newly validated housekeeping genes [54] could have been used for gene expression normalization.

### Brain endothelial cell surface receptors expression

**Figure 5:**
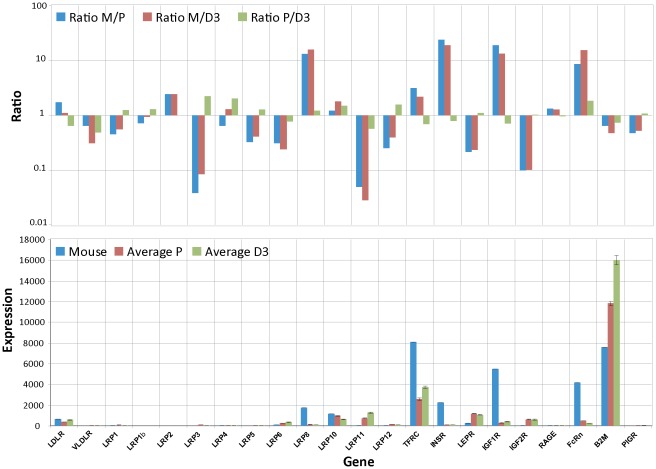
Differences in expression levels of surface receptors between the cultured BECs and freshly isolated BECs. The RPL4 normalized mouse expression values (Mouse) (RPL4 normalized) are compared to the also RPL4 normalized expression values of hpBECs (Average P) and hCMEC/D3 (Average D3). The lower graph shows the absolute expression levels for each cell type. The upper graph shows the ratio between RPL4 normalized mouse BECs and hpBECs (Ratio M/P), the ratio between RPL4 normalized mouse BECs and hCMEC/D3 (Ratio M/D3) and the ratio between hpBECs and hCMEC/D3 (Ratio P/D3). Four genes that are expressed in much lower levels in both the hpBECs and the hCMEC/D3 cell line are Lrp8, IR, IGF1R and FcRn.

Another class of proteins important for BBB function are the cell surface receptors e.g the Low Density Lipoprotein (LDL) receptor family. These receptors are able to bind and internalize a plethora of ligands and therefore play an important role in diverse physiological processes. Some of the members have been described to play a unique role in the transcytosis of various ligands at the BBB. The expression levels of the LDL receptor family members are shown in **Figure 5** together with two other key endocytic receptors, transferrin receptor (TfR) and the insulin receptor (IR). The expression profile for these receptors is significantly different in the *in vitro* BEC systems compared to the expression levels of freshly prepared BECs [16] (**Figure 5**). Four receptor genes that are higher expressed *in vivo* are LRP8, IR, IGF1R and FcRn. One receptor expressed at high levels *in vivo* as well as in the cultured BECs is the TfR (**Figure 5**). This receptor has already been shown to be expressed on the hCMEC/D3 cells by FACS [55]. LRP1 and RAGE have been described to be involved in amyloid-β transport across the BBB [56]. LRP1 has also been described to be a transporter for various proteins and peptides over the BBB [57]. However, both receptors are expressed at low levels in both the cultured human and the freshly prepared mouse BECs (**Figure 5**).

### Brain endothelial transporter expression

**Figure 6:**
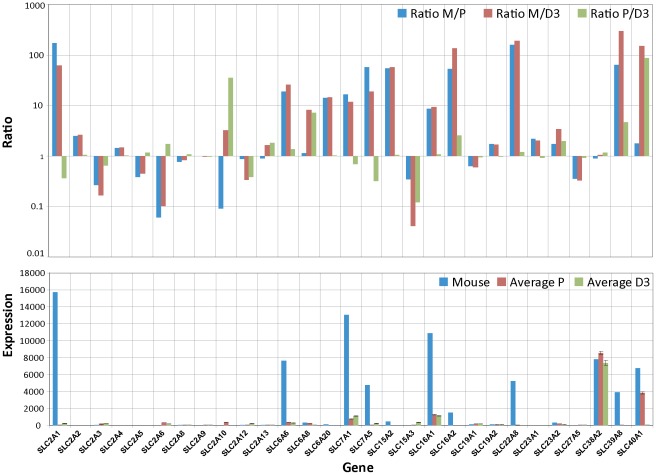
Differences in expression levels of SLC transporter between cultured BECs and freshly isolated BECs. The RPL4 normalized mouse expression values (Mouse) are compared to the also RPL4 normalized expression values of hpBECs (Average P) and hCMEC/D3 (Average D3). The lower graph shows the absolute expression levels for each cell type. The upper graph shows the ratio between RPL4 normalized mouse BECs and hpBECs (Ratio M/P), the ratio between RPL4 normalized mouse BECs and hCMEC/D3 (Ratio M/D3) and the ratio between hpBECs and hCMEC/D3 (Ratio P/D3). For instance genes that are expressed in much lower levels in both hpBECs and the hCMEC/D3 cell line are GLUT1, MCT8 and OAT3.

**Figure 7:**
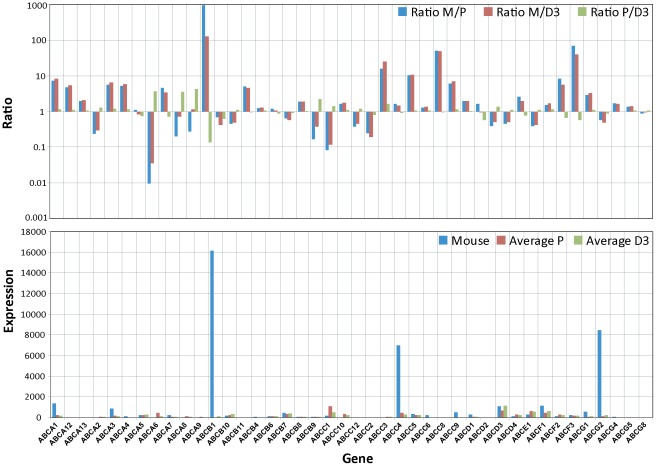
Differences in expression levels of ABC transporter between cultured BECs and freshly isolated BECs. The RPL4 normalized mouse expression values (Mouse) are compared to the also RPL4 normalized expression values of hpBECs (Average P) and hCMEC/D3 (Average D3). The lower graph shows the absolute expression levels for each cell type. The upper graph shows the ratio between RPL4 normalized mouse BECs and hpBECs (Ratio M/P), the ratio between RPL4 normalized mouse BECs and hCMEC/D3 (Ratio M/D3) and the ratio between hpBECs and hCMEC/D3 (Ratio P/D3). Three genes that are expressed in much lower levels in both the hpBECs and the hCMEC/D3 cell line are Pgp1, MRP4 and MDRA1.

To further investigate the status of the both BEC lines we next examined the expression profiles for the two important transport families, the solute carrier (SLC) and the ATP-binding cassette (ABC) transporters [58], [59]. These two classes of BBB proteins are responsible for the transfer or exclusion of nutrients and toxic agents. Notably, the majority of the substrates for these transporters are small molecules, including a variety of dipeptides. An overview of the SLC family gene expression profile is displayed in **Figure 6** and again compared to the freshly prepared BECs from mouse [16]. The data is displayed using the same normalization procedure using the housekeeping gene RPL4. The highly expressed transporters identified using the mouse BECs such as Glut1 (slc2a1), MCT1 (slc16a1), MCT8 (slc16a2), TauT (slc6a6), CAT1 (slc7a1) and LAT1 (slc7a5) are expressed at very low levels in both cultured human BECs. This is consistent with the previous data on the TJs and receptors where BBB specific genes are significantly reduced in expression levels in the cultured human BECs. Thus, SLC family members that are known to be involved in vital functions at the BBB and are expressed at high levels at the BBB, show a more average expression level in the cultured human BECs. We performed the same analysis for the ABC transport family and strikingly the genes which are highly expressed in the mouse BECs were strongly reduced in the both cultured human BECs (**Figure 7**). The typical ABC transporters at the BBB, such as Pgp, (ABCB1), MRP4 (ABCC4) and BCRP (ABCG2), were almost reduced to undetectable levels (**Figure 7**). Importantly, the change in expression levels within these two families of transporters are affected in both directions (**ratio plot in** **Figure 6** **and** **7**), indicating an asymmetric perturbation of the transcriptional regulation machinery. Cultured cells fail to maintain high levels of key BBB transporters. Taken together, it appears that the unique expression pattern of these typical BBB transporter family members disappears when BECs are isolated from their natural environment.

## Discussion

In this study we present evidence that those genes in BECs that are highly expressed and described to be important for ensuring BBB-like properties are particularly affected by culturing the cells in isolation. This is most likely due to the loss of their native environment within the neurovascular unit (NVU).

The information was gathered by investigating how BECs respond to culture conditions without their in vivo surrounding. Astrocytes, pericytes and neurons normally interact directly or indirectly with the endothelium within the intricate structure of the NVU [3], [4], [5], [6]. For instance, it has been recently shown that pericytes can have a direct effect on endothelial cell properties in vivo, where reduced expression of pericytes increased the transcellular transport across the endothelial cells [60], [61]. Astrocytes have previously been described to modulate BBB permeability [38] and recently it has been shown that astrocytes have a pivotal role in dynamic signaling within the NVU related to regulation of cerebral blood flow [62]. In this study we used two independently derived human BECs, one of primary source (hpBECs) and the other an immortalized cell line (hCMEC/D3). We show that these two BECs have a very similar global gene expression profile (R^2^ = 0.91, p<1.68 ⋅ 10^−6^). Importantly, both cell types are pure endothelial cell populations based on the presence of typical endothelial cell surface markers (**Figure 1a–1c**).

Some of the major differences in gene expression can be attributed to the immortalization procedure, where genes know to be affected by SV40 large T antigen integration [30] have also been altered in the hCMEC/D3 cell line. As a consequence of the immortalization procedure genes belonging to RNA processing, DNA repair, immune and virus response and mitosis are highly and significantly up-regulated in the hCMEC/D3 cell line (**Figure 1f** **, Table S2 and S7**). Genes that are expressed at higher levels in the hpBEC based on the gene set enrichment analysis belong to vesicle transport, endothelial migration and catabolic/metabolic processes, possibly indicating a deficiency of these properties in the hCMEC/D3 cell line. Genes related to the IFN signaling pathway have higher expression levels in hCMEC/D3 (**Figure 2a**). The expression of MHC class I and II genes are significantly higher in the hCMEC/D3 cell line which is confirmed by FACS analysis (**Figure 2c–2e**). The hpBECs are more responsive towards IFN stimulation whereas the hCMEC/D3 cells are already in an activated state (**Figure 2d and 2e**). This indicates that immunological responses could be significantly affected in the hCMEC/D3 cell line as a result of the immortalization procedure. The hCMEC/D3 cells proliferate faster (**Figure 1d**) and persist in a confluent state for a longer period (**Figure 1d**). We speculate that the up regulated expression of anaphase and mitosis related genes, identified by gene set enrichment analysis (**Figure 1f**), alter the growth characteristics of the hCMEC/D3 cells compared to the hpBECs. Thus, this immortalized cell line should be used with caution, especially if immunological processes are being investigated.

The coefficient determination (R^2^) with and without astrocyte co-culturing was 0.998 (p<4.18 ⋅ 10^−8^) and 0.997 (p<5.88 ⋅ 10^−8^) for the hpBECs and the hCMEC/D3 cell line, respectively. Overall, this demonstrates that none of the BECs responded strongly to astrocytic co-culturing at the gene expression level (**Figure 3a and 3b**). The data in the figures illustrate the low number of genes that were affected by the presence of astrocytes. This is in agreement with the unaltered functional properties of these BECs, such as TEER values and paracellular permeability in co-culture with astrocytes. Our transcriptional analysis of the BECs in presence of astrocytes was performed in a non-contact arrangement to avoid any risk of contamination by astrocytic mRNA. This experimental set up lacking the direct contact with the astrocytes might explain the low responsiveness of the BECs at transcriptional level. However, when using a contact astrocyte co-culturing set up, no significant change could be detected in either TEER (BECs+HA = 10–30 Ω/cm^2^) or paracellular permeability (Pe values: hCMEC/D3 = 3.5±0.2 ⋅ 10^−5^ cm/sec, hCMEC/D3 + HA 3.3±0.3 ⋅ 10^−5^ cm/sec, hpBECs = 3.4±0.4 ⋅ 10^−5^ cm/sec, hpBECs+HA = 3.7±0.4 ⋅ 10^−5^ cm/sec). There are reports describing the effects of astrocytic co-culture in which the focus of the study was on TJ properties. One recent study using an immortalized mouse brain endothelial cell line in co-culture with primary rat astrocytes showed a small but non-significant increase in TEER but no changes in gene expression of various TJ proteins [63]. Another recent investigation using primary porcine brain endothelial cell showed that co-culturing with a mixture of primary glial cell from rat could have a profound effect on the endothelial cell properties, such as increased TEER values and claudin-5 expression [42]. In addition, recently it was shown that claudin-5 was significantly increased in three different *in vitro* BBB models by the influence of astrocytes [**64**]. We were unable to identify changes in gene expression related to TJs formation in these two BECs using a non-contact astrocyte co-culturing set up.

However, we identify other genes that were significantly influenced by the presence of astrocytes, and importantly some of them were changed in both BECs (**Figure 3c**). Interestingly, among these 140 genes that were significantly down-regulated in both BECs in presence of astrocytes, many could be assigned to genes involved in cell-cell adhesion, cellular extravasation and cell migration GO categories. A closer statistical investigation of these gene sets as whole indeed revealed a significant down-regulation of their members in both BECs when co cultured with astrocytes. For instance, E-selectin (SELE) together with vascular cell adhesion molecule 1 (VCAM-1), intracellular cell adhesion molecule 1 (ICAM-1) and leucocyte surface antigen CD47 (MER6), all part of the signaling platform for EC interaction with leukocytes [65], are included in these common genes which were affected by astrocytic co-culturing. Possibly, this could be related to the regulation of leukocyte migration and extravasation into the CNS [65]. Leukocyte recruitment by ECs is regulated by the expression of surface adhesion molecules, which are responsible for decelerating and capturing circulating immune cells. The extent to which this occurs is governed by the degree of expression and activity of these surface adhesion molecules and the activation state of the leukocytes. Based on these data we speculate that astrocytes might affect not only the BBB permeability but also the expression of adhesion molecules, inducing a low expression and activation state of adhesion molecules on BECs and thereby be directly involved in the regulation of the immune surveillance status of the CNS. Thus, astrocytes seem to have a specific function in cerebral vessels and capillaries regulating extravasation of blood cells over the BBB, this is not the case in the peripheral vasculature which lacks astrocytic cells. These data are in agreement with a very recent publication showing that sonic hedgehog which is produced by astrocytes promotes immune quiescence of BBB ECs by decreasing expression of proinflammatory mediators and the adhesion and migration of leucocytes [66]

**Figure 8:**
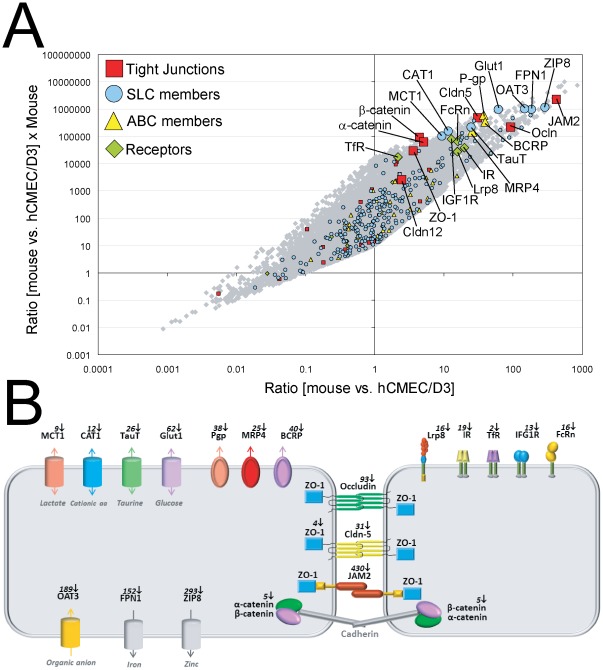
Key BBB genes are expressed at low levels in cultured human BECs. *(* ***A*** *)* All genes for mouse BECs versus hCMEC/D3 are displayed as grey dots. Tight junction genes (red square), SLC members (blue dot), ABC members (yellow triangle) and the surface receptors (green diamond) are highlighted. The x-axis shows the ratio between mouse and hCMEC/D3 and the y-axis shows the ratio between mouse BECs and hCMEC/D3 multiplied by the expression levels in mouse BECs. Genes in the upper right corner are therefore highly expressed in mouse BECs and much more in comparison to the hCMEC/D3 cell line. The graph shows that all key BBB genes are expressed at lower levels in the human cell line hCMEC/D3 (enlarged symbols). A similar result was obtained when comparing the mouse expression data to the hpBECs. *(* ***B*** *)* A schematic representation of the key genes identified in this study to have low level of expression in the hCMEC/D3 cells. The picture illustrates the spatial location of the BBB genes on BECs and the fold reduction in expression compared to mouse BECs.

Certain key conclusions made in this paper are based on comparison of the isolated human BECs transcriptome data with the data generated using freshly purified BECs from mouse [16]. This is an important procedure since the mouse data can be assumed to represent essential *in vivo* gene expression patterns important for unique and distinctive BEC properties. We believe this is a valid approach despite the species differences because the established housekeeping gene RPL4 was used for normalization of the probe signals [54]. In addition, by comparing the top 14 candidate housekeeping genes for human and mouse the average ratio is 1.12 with a standard deviation of 0.518 (**Figure S4**). This tight agreement between these housekeeping genes was obtained even though these two data sets were obtained in two independent studies. This is far below the expression differences we are using in our discussions and conclusions regarding BBB specific genes. Moreover, the human and mouse data comparison is also consistent with an unbiased bidirectional change in expression pattern within a family of genes with similar biological function, such as for the SLC family depicted in **Figure 8a**. It is also interesting to note that the correlation between the mouse and human data is high, the coefficient of determination between mouse and hpBECs and the hCMEC/D3 cell line is 0.482 (p<7.74 ⋅ 10^−5^) and 0.455 (p<2.50 ⋅ 10^−9^), respectively. This means that about half of the variability in the human cells can be explained by the variability in the mouse BECs. Potentially, the half with global cross-species correlation could potentially represent transcripts which are not imposed by the natural *in vivo* environment. While the other half of the gene set where no clear correlation was obtained could belongs to transcripts that are directly regulated within the NVU surroundings where most of these genes belong to BBB specific transcripts discussed in this paper. Recently, an extensive brain gene expression profiling between mouse and human was undertaken. This cross-species analysis showed that gene expression is significantly preserved between the two species (R = 0.60; p<10^−400^) [17].

A particular focus of this study was the understanding of TJ properties at transcriptional level in order to elucidate some of the key genetic attributes necessary for proper BBB function. Our transcriptome data on the hCMEC/D3 cell line and the hpBECs are in agreement with relative high paracellular leakiness and low TEER values. In our hands, the hCMEC/D3 cells have a TEER value in the region of 15–30 Ω/cm^2^ which is in agreement with published values [15] and is similar to values obtained in peripheral capillaries. Similar low TEER values were also obtained for the hpBECs. These numbers should be compared to *in vivo* values where the resistance has been estimated to be approximately 1500–8000 Ω/cm^2^ [67], [68], [69]. Certain members of the claudin [52] family and occludins [70] have been described to regulate the diffusion of certain ions between ECs. Claudin expression within the TJs seems to determine the ion selectivity of the paracellular diffusion [52], meaning that not only the existence of certain TJ proteins are important but also their expression levels in relation to other TJ proteins. Our data shows that occludin and claudin-5 are expressed at very low levels in the cultured human BECs. These interpretations are based on mRNA quantification and not on directly measured protein levels which could be misleading. However, a recent investigation on thousands of genes in mammalian cells shows a good correlation (R^2^ = 0.41) between mRNA and protein levels [71]. These two TJ proteins have been described to play a pivotal role in maintaining low paracellular permeability at the BBB [51], [70], [72], [73]. Thus, the low expression of claudin-5 and occludin are probably one of the reasons for the low TEER values in these two cell lines. However, this might not be corrected by a simple overexpression of only these two TJ proteins. Correction of the levels of all TJ protein may be needed to establish functionalities resembling *in vivo* properties. Our gene expression analysis and the comparison with the mouse transcriptome data indicate that the absolute levels and the relative expression of some important TJ proteins in hCMEC/D3 cell line and hpBECs are altered (**Figure 4**). The precise expression profile of the TJ proteins in BECs is probably dictated by their environment and this regulatory mechanism is altered when the BECs are cultured in isolation. Recently, attempts have been made to develop the properties of the hCMEC/D3 cell line by co-culturing with astrocytes and pericytes [74] but so far the improvement has been small. Thus this research area needs additional attention to ensure further advancements.

One important transport mechanism for proteins across the BBB is thought to be facilitated by receptor-mediated transport (RMT) [75]. The transferrin and insulin receptors have been shown to transport cargo across the BBB using a transcytosis mechanism [76]. These two receptors are expressed in both the hCMEC/D3 cell line and the hpBECs based on our gene expression data (**Figure 5**) and is in agreement with published data [55]. However, other described transporters such as LRP1 and RAGE are expressed at very low levels in both the two cultured human cell lines as well as in the freshly prepared mouse BECs. The ability of these two receptors to transport amyloid-β over the BBB is currently under investigations [77], [78]. Interestingly, there are four additional receptors with high transcriptional levels in BECs based on the mouse *in vivo* data (Lrp8, IR, IGF1R and FcRn) which fail to be maintained at high levels in the two cultured human cell lines (**Figure 5**). Possibly, as seen for the TJ proteins, the transcription of these receptors is specifically regulated by the neighboring cells within the NVU.

Cell surface transporters are the gatekeepers for all cells and organelles, controlling uptake and efflux of crucial compounds such as sugars, amino acids, nucleotides, inorganic ions and drugs. These transporters are especially important for BBB function since they regulate homeostasis inside the brain by selectively determining what should be permitted to enter or exit the brain. The two most important transport families are the SLC and ABC transporters [79]. Strikingly, typical BBB transporters that are highly expressed in the freshly prepared mouse BECs are in general expressed at low levels in the two human cell lines (**Figure 6** **and** **7**) This is clearly seen within the SLC family where there is low expression of Glut1 (SLC2A1), MCT1 (SLC16A1), MCT8 (SLC6A2), TauT (SLC6A6), CAT1 (SLC7A1) and LAT1 (SLC7A5). In the ABC family, members like Pgp (ABCB1), MRP4 (ABCC4) and BRCP (ABCG2) are also affected in a similar manner. Thus, our data strongly suggest that transporters that are highly expressed *in vivo* fail to be preserved in ECs *in vitro*.

Some of the key findings in this paper are summarized in **Figure 8** where the expression levels of all genes are compared between the purified mouse BECs with the human immortalized cell line hCMEC/D3. The distribution of the ratio in expression levels among genes between the two BECs is centered at one, demonstrating that equal amounts of genes are either up or down regulated in the hCMEC/D3 cell line compared to the mouse BECs. This is also the case when analyzing expression levels of particular families of genes, indicating that the changes identified are not due to a systematic loss of expression of entire family of genes. For instance the expression of the large SLC family is evenly distributed amongst all genes, which is also the case for the claudin family members (**Figure 8**). However, genes that have been described to possess a unique BBB function are all expressed at very low levels in the hCMEC/D3 cell line (**genes indicated in** **Figure 8**). The low expression levels of these characteristic BBB genes were also seen for the hpBEC, suggesting that this massive reduction in expression of typical BBB genes could be a general phenomenon when BECs are studied in isolated cultures. The mere presence of a particular protein detected by immunocytochemistry is no guarantee for correct cellular function. The absolute level of expression is very likely to influence the cellular phenotypic properties.

In general, the findings of our study illuminate the need for improved *in vitro* BBB models. Especially when complex biological mechanisms such as transcellular transport, intracellular sorting of proteins and extravasation of cells are being investigated. Detailed characterization is necessary for better understanding of the data generated in the *in vitro* models and its relevance. Importantly, BECs are heavily influenced by their native environment and this has to been taken into account when designing the right conditions for a predictive *in vitro* BBB model.

## Materials and Methods

### Cell lines and culture conditions

Immortalized human capillary endothelial cells (hCMEC/D3) were obtained under license from INSREM France (Weksler BB, et al. (2005) Blood-brain barrier-specific properties of a human adult brain endothelial cell line. Faseb J 19: 1872–1874) and the human primary cerebral microvascular endothelial cells (hpBECs) were purchased from the Applied Cell Biology Research Institute (Kirkland, WA). The human primary astrocytes (HAs) were purchased from ScienCell Research Laboratories (San Diego, CA). The hCMEC/D3 cells used for the experiments were between passage (p) 27 and 32. The hpBECs (p2) and the HAs (p3) were grown for maximal 2 additional passages. All culture ware (BD Falcon) and transwell filters (Millipore) (pore size 0.4 µm, high density pores) were coated with rat tail collagen type I solution (BD Bioscience) at a concentration of 10 ug/cm^2^ for 1 hour at 37°C according to the manufactures instructions. All endothelial cells were grown in EBM-2 Medium (Lonza Bioscience) supplemented with EGM-2 containing hFGF-B, VEGF, R3-IGF, ascorbic acid, hEGF, hydrocortisone and heparin (Lonza Bioscience). For functional assays the cells were grown in a growth factor depleted EBM-2 medium containing 3% human serum (Blood Bank, Basel, Switzerland) and 0.55 µM hydrocortisone (Sigma) in the following referred as the resting medium. Cells were cultured in the incubator at 37°C with 5% CO_2_, 95% fresh air and saturated humidity. Cell culture medium was changed every 3 days.

### Co-cultivation with human astrocytes

5×10^4^ HAs/cm^2^ were seeded in astrocytic medium (ScienCell Research Laboratories), containing astrocytic growth supplements (ScienCell Research Laboratories) onto poly-L-lysine (Sigma) coated 12 well plates. 3 days after seeding, after the HAs were confluent, freshly collagen coated transwell filters were transferred to the 12 well plates and the culture medium was replaced by resting medium. 5×10^4^ of either hCMEC/D3 or hpBECs were subsequently seeded onto the transwell filters and co-cultured for 3 days.

### Interferon stimulation

Confluent hpBECs and hCMEC/D3 were treated with either human recombinant IFNγ (R&D Systems) or human recombinant IFNα (Roferon-A, Roche, Switzerland) (100 U/ml each) for 16 hours at 37°C before they were Flow Cytometry analysed.

### Real time impedance measurement

Cell growth behavior was continuously monitored every 15 minutes for 7 days using a Real Time Cell Analyzer (xCeLLigence, Roche). For time-dependent cell response profiling, 100 µl of cell culture medium was added to the collagen I coated 96 well E-plates to obtain background reading followed by the addition of 100 ul of cell suspension. The E-plates containing the cells were allowed to incubate at RT for 15 minutes and placed on the reader in the incubator for continuous recording of impedance as reflected by cell index (CI). The CI curves are displayed as the average of 4 replicates +/− standard deviation. All the data was normalized to the first impedance background measurement.

### RNA extractions

For microarray analyses endothelial RNA was isolated from four individual transwell filters per experimental setting: hCMEC/D3, hCMEC/D3 + HA, hpBECs and hpBECs + HA. RNA extractions were performed using RNeasy Mini Kit (Qiagen) according to the manufacturer's instructions. Quality and quantity of all isolated RNAs were determined with a NanoDrop ND 1000 (NanoDrop Technologies) and a Bioanalyzer 2100 (Agilent). The RNA integrity numbers in all cases were from 8.3 to 10 indicating minimal RNA degradation.

### Microarray procedures and data processing

Whole genome expression profiles were generated for all samples plus two control universal human reference RNA (Stratagene) samples. Four Affymetrix chips were used for microarray hybridization of each condition (i.e., cell-type, co culturing). 100 ng of total RNA from each sample were used to generate biotinylated aRNA, using the Affymetrix GeneChip 3′ IVT Express kit according to manufacturer's instructions (Affymetrix Inc, CA, USA). 16 hours incubation was used for the in vitro transcription reaction. The cRNA samples were hybridized overnight to Affymetrix U133 Plus 2.0 full genome oligonucleotide arrays and then stained with Streptavidin-Phycoerythrin according to the manufacturer's instructions (Affymetrix). Arrays were scanned using a GeneChip Scanner 3000 (Affymetrix) and signal intensities were calculated automatically by Affymetrix GeneChip Command Console. Microarray data were analyzed with R (Version 2.11.1) using the affy, affyPLM, genefilter, and limma Bioconductor packages. All arrays passed a QC inspection and showed similar background values and distributions of signal intensities. Preprocessing (probe summarization, background correction and quantile normalization) was done with the RMA algorithm. Before examining differential expression, spike-in probe sets were removed, along with poor quality probe sets. These were defined as probe sets with three or more probes that were poor (e.g. the probe sequence was not unique, based on more recent public annotations as well as internal annotations). Finally, probe sets with low expression (14 or more samples with log_2_ expression <5) across samples were removed [80] [81], leaving 21460 probe sets in the final analysis. Differential expression was assessed using the limma package, and multiple testing was taken into account by using the false discovery rate (FDR) rather than unadjusted p-values. Gene set enrichment analysis (GSEA; [82]) was used to look for enrichment of differentially expressed genes between the two cell types, based on Gene Ontology (GO) biological process terms. Gene sets smaller than 10 or greater than 400 were not included. If a gene had multiple probe sets targeting it, the probe set with the highest mean expression (across all samples) was used. For the human (*in vitro*) versus mouse (*in vivo*) comparison, log_2_ expression values were normalized to RPL4, a ribosomal protein that has been shown to have relatively stable expression across species [54]. The difference between the mouse and human values were used as input for the GSEA algorithm from the Broad Institute [81].

### Flow Cytometry Analysis

Confluent resting Brain Endothelial Cells (BECs) or HAs monolayers were detached from the transwell filters with Trypsin-EDTA (Gibco). Single cell suspension was subsequently incubated in resting medium at 37°C for 1 hour prior to the staining. All antibodies were directly labeled and purchased from BD Pharmingen. After the staining the cells were washed twice with stain buffer (BD Pharmingen) and analyzed using a Guava easyCyte flow cytometer (Millipore). For surface staining, 1×10^6^ cells were pelleted and incubated for 45 minutes at 4°C with the following antibodies: anti-CD31-PE (WM59), anti-CD34-APC (581/CD34), anti-CD105-Alexa_647_ (266), anti-CD54-PE (HA58), anti-HLA-ABC-FITC (G46-2.6), anti-HLA-DR,DP,DQ-FITC (Tu39), IgG1-PE (MOPC-21), IgG1-FITC (MOPC-21), IgG1-APC (MOPC-21), IgG1-Alexa_647_ (MOPC-21) and IgG2a-FITC (G155-178). For intracellelular staining 1×10^6^ pelleted HAs were fixed (BD Cytofix) for at 37°C for 10 minutes, permeabilized (BD Phosflow Perm III) on ice for 30 minutes and incubated with the following: anti-GFAP-Alexa_647_ (1B4) and IgG2b-Alexa_647_ (27–35).

## Supporting Information

Figure S1 **Phase contrast microscopic imaging on hpBECs and hCMEC/D3 cells.** Phase contrast microscopy of confluent hpBECs and hCMEC/D3 cells, at 3 days post seeding on collagen coated plastic dish (bar = 50 µm). The pictures illustrate the typical phenotype of an endothelial cell monolayer in which the cells partially aligned their grow position to each other.
(TIF)

Figure S2 **Human astrocytes analysis with FACS using GFAP expression as a marker.** Flow cytometry analysis of confluent human Astrocytes (HAs) 3 days post seeding, seeded on poly-L-lysine coated plastic dish. The intracellular staining was done with the indicated antibody (green histogram) or a relevant isotype control (tinted histogram). GFAP expression level in one of three similar experiments is presented. A minor fraction of the cells showed a high expression level of the GFAP whereas the vast majority of HAs express GFAP at intermediate levels.
(TIF)

Figure S3 **Brain endothelial cells in astrocyte co-culturing alter ICAM-1 expression levels.** Flow cytometry analysis of confluent hCMEC/D3 cells co-cultured with human Astrocytes for 3 days. The mean fluorescence intensity (MFI) of ICAM-1 surface expression with (green histogram) and without (black histogram) astrocytic co-culturing are shown. The grey histogram shows the background binding of the relevant isotype control antibody on hCMEC/D3 cells without co-culturing. One of three similar experiments is displayed.
(TIF)

Figure S4 **Gene expression analysis of housekeeping genes from human and mouse brain endothelial cells.** *(* ***A*** *)* The variation of proposed housekeeping genes between human and mouse probe sets after normalization using the Rpl4 gene showing low deviation from a ratio of one. The average ratio for all 14 housekeeping genes are 1.12 with a standard deviation of 0.518. *(* ***B*** *)* Comparison of gene expression between hCMEC/D3 cells and mouse pBECs. The data is represented as a dot plot on a log_2_ scale, where each point represents a probe set on the gene chip. Red and blue dots indicate probe sets, which have higher expression in hCMEC/D3 (red) or higher expression in mouse pBECs (blue). Grey dots show the expression respectively difference of expression of all 14 housekeeping genes between the species.
(TIF)

Table S1 **All average expression data.**
(XLS)

Table S2 **hCMEC D3 vs. hpBECs gene sets.**
(XLS)

Table S3 **IFN signaling reactomes hCMEC D3 vs. hpBECs.**
(XLS)

Table S4 **BECs + HA, gene sets.**
(XLS)

Table S5 **BECs + HA, genes in hCMEC D3 and hpBECs.**
(XLS)

Table S6 **hCMEC D3 + HA, gene sets.**
(XLS)

Table S7 **Mouse pBECs vs. hCMEC/D3, gene sets.**
(XLS)
