# Supplementary figures and images for: Transcriptional Profiling of Human Brain Endothelial Cells Reveals Key Properties Crucial for Predictive In Vitro Blood-Brain Barrier Models
Source: PLoS One. 2012 May 31;7(5):e38149. doi: 10.1371/journal.pone.0038149 (PMC3364980; doi:10.1371/journal.pone.0038149)

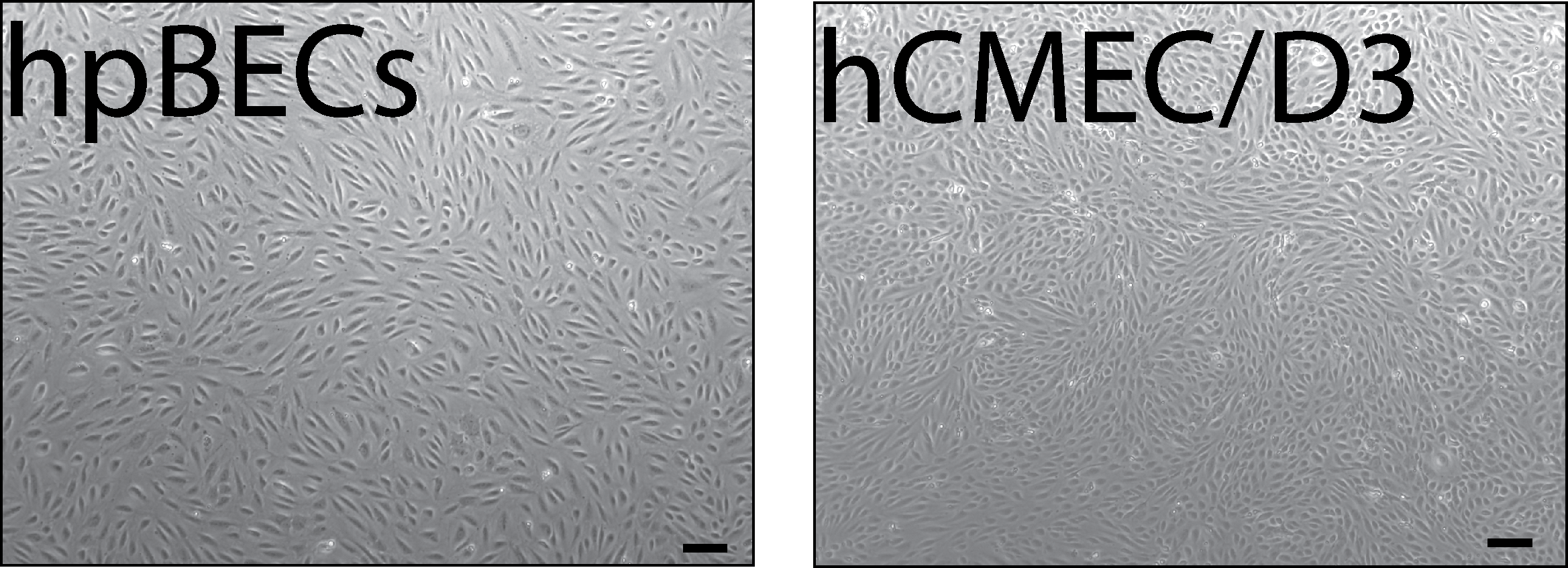

Supplement: Figure S1 — Phase contrast microscopic imaging on hpBECs and hCMEC/D3 cells. Phase contrast microscopy of confluent hpBECs and hCMEC/D3 cells, at 3 days post seeding on collagen coated plastic dish (bar = 50 µm). The pictures illustrate the typical phenotype of an endothelial cell monolayer in which the cells partially aligned their grow position to each other. (TIF) [file pone.0038149.s001.tif]

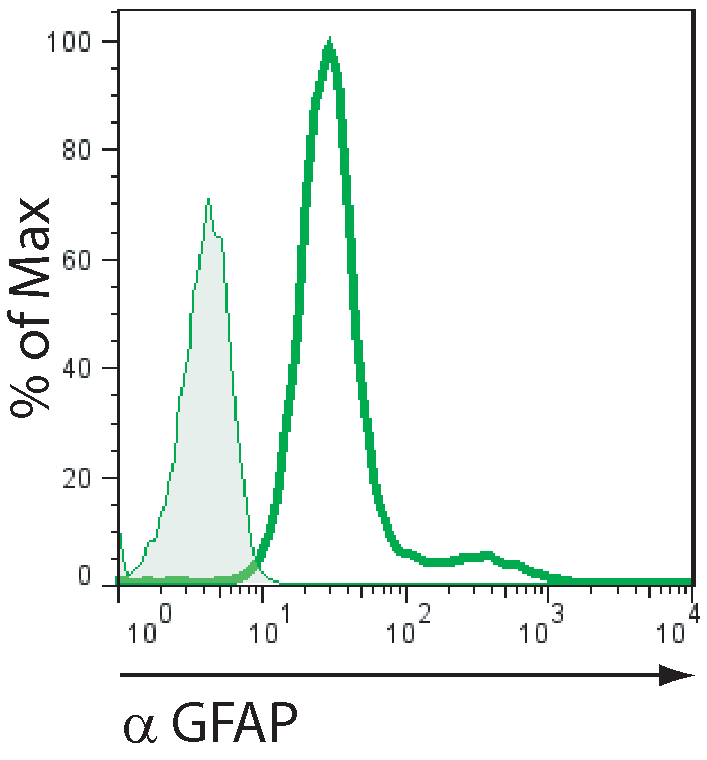

Supplement: Figure S2 — Human astrocytes analysis with FACS using GFAP expression as a marker. Flow cytometry analysis of confluent human Astrocytes (HAs) 3 days post seeding, seeded on poly-L-lysine coated plastic dish. The intracellular staining was done with the indicated antibody (green histogram) or a relevant isotype control (tinted histogram). GFAP expression level in one of three similar experiments is presented. A minor fraction of the cells showed a high expression level of the GFAP whereas the vast majority of HAs express GFAP at intermediate levels. (TIF) [file pone.0038149.s002.tif]

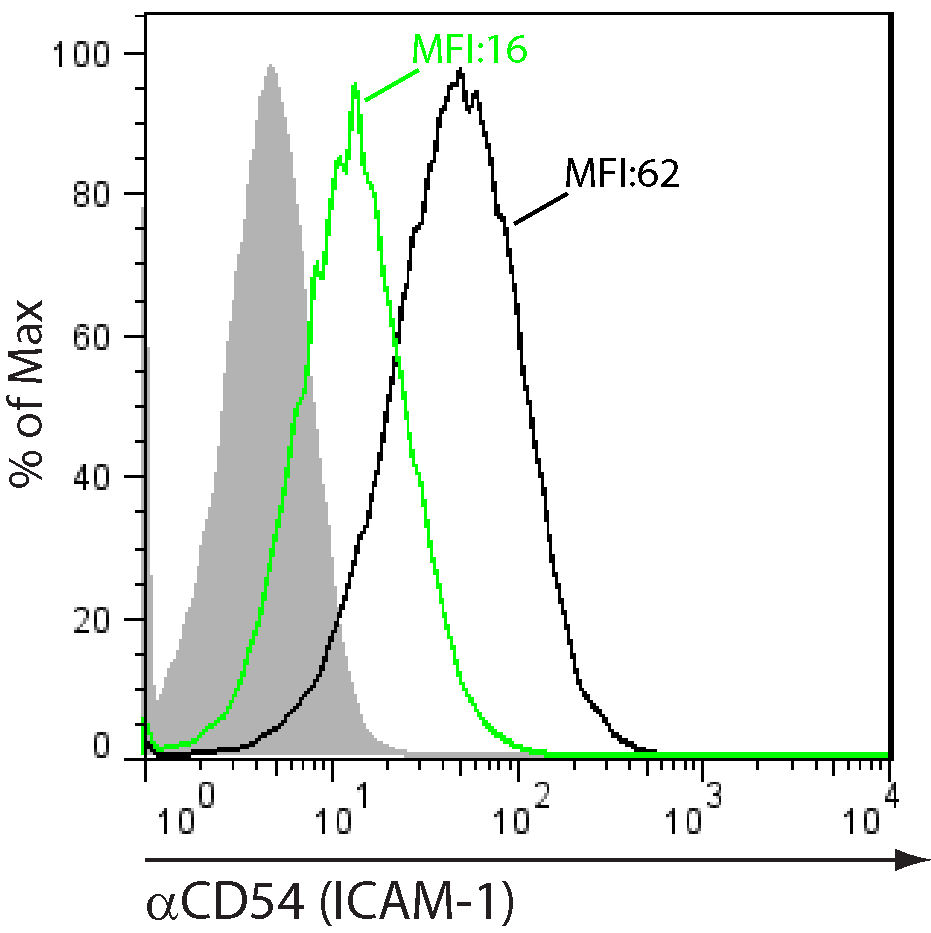

Supplement: Figure S3 — Brain endothelial cells in astrocyte co-culturing alter ICAM-1 expression levels. Flow cytometry analysis of confluent hCMEC/D3 cells co-cultured with human Astrocytes for 3 days. The mean fluorescence intensity (MFI) of ICAM-1 surface expression with (green histogram) and without (black histogram) astrocytic co-culturing are shown. The grey histogram shows the background binding of the relevant isotype control antibody on hCMEC/D3 cells without co-culturing. One of three similar experiments is displayed. (TIF) [file pone.0038149.s003.tif]

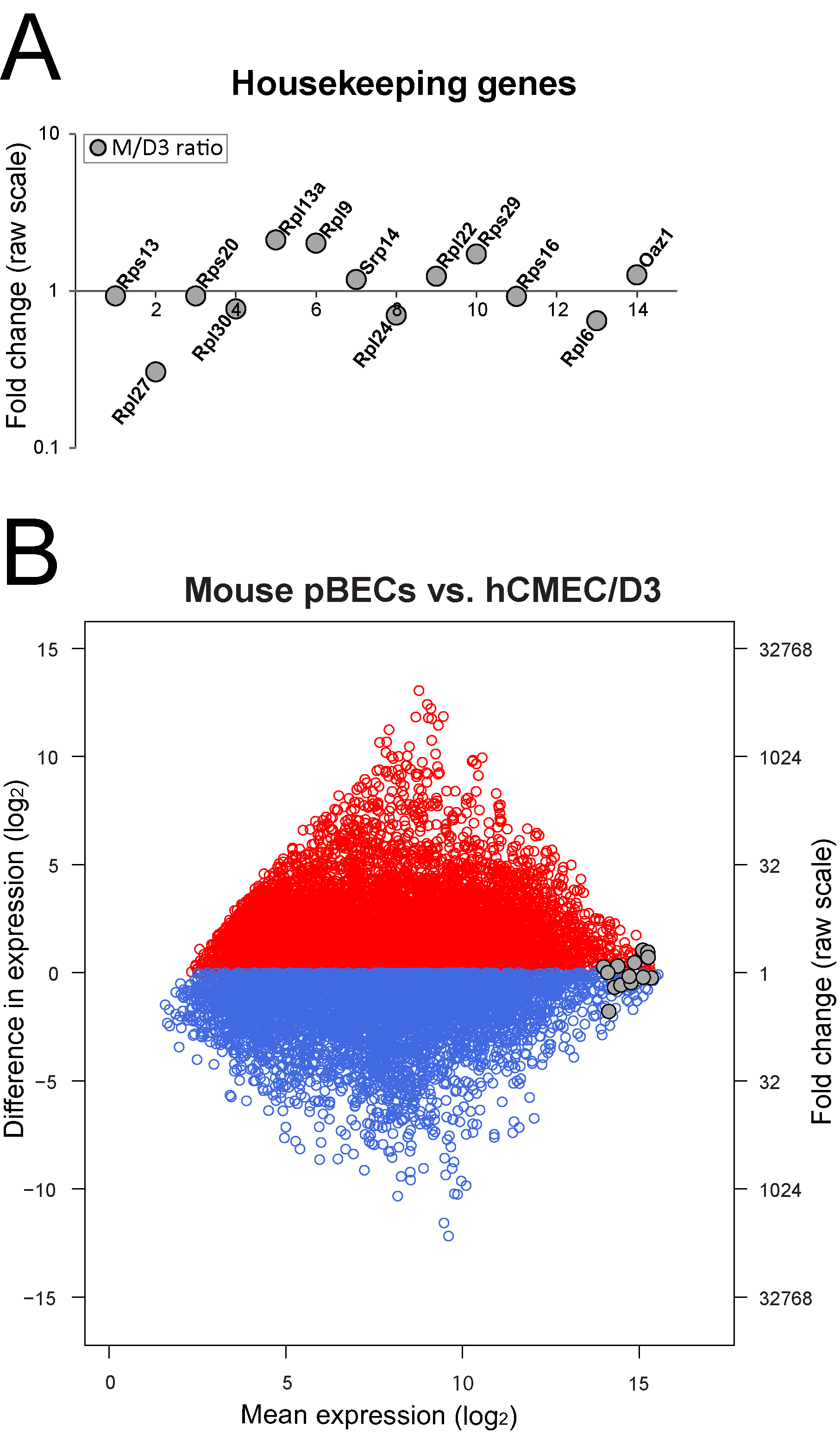

Supplement: Figure S4 — Gene expression analysis of housekeeping genes from human and mouse brain endothelial cells. ( A ) The variation of proposed housekeeping genes between human and mouse probe sets after normalization using the Rpl4 gene showing low deviation from a ratio of one. The average ratio for all 14 housekeeping genes are 1.12 with a standard deviation of 0.518. ( B ) Comparison of gene expression between hCMEC/D3 cells and mouse pBECs. The data is represented as a dot plot on a log2 scale, where each point represents a probe set on the gene chip. Red and blue dots indicate probe sets, which have higher expression in hCMEC/D3 (red) or higher expression in mouse pBECs (blue). Grey dots show the expression respectively difference of expression of all 14 housekeeping genes between the species. (TIF) [file pone.0038149.s004.tif]
